# Retrieval-Induced Forgetting in a Pentylenetetrazole-Induced Epilepsy Model in the Rat

**DOI:** 10.3390/brainsci8120215

**Published:** 2018-12-05

**Authors:** Ahmad Almahozi, Maan Alsaaid, Saeed Bin Jabal, Amer Kamal

**Affiliations:** Physiology Department, College of Medicine and Medical Sciences, Arabian Gulf University, P.O. Box 26671, Manama 1111, Bahrain; maan.alsaaid@gmail.com (M.A.); saeed701ys@hotmail.com (S.B.J.); amerha@agu.edu.bh (A.K.)

**Keywords:** retrieval-induced forgetting, epilepsy, kindling, pentylenetetrazole, inhibition, memory, rat, spontaneous object recognition

## Abstract

The selective retrieval of some information may lead to the forgetting of related, but non-retrieved information. This memory phenomenon is termed retrieval-induced forgetting (RIF). Active inhibition is thought to function to resolve interference from competing information during retrieval, which results in forgetting. Epilepsy is associated with impaired inhibitory control that contributes to executive dysfunction. The purpose of this study is to investigate whether rats in a kindling model of epilepsy demonstrate normal levels of RIF. Rats were divided into two groups: saline and kindling. Pentylenetetrazole was injected intraperitoneally until the rats kindled. RIF was tested using a modified version of the spontaneous object recognition test, consisting of a sample phase, retrieval or interference phase, and a test phase. Exploration time for each object was analyzed. RIF was demonstrated in the saline group when rats subjected to the retrieval phase failed to discriminate between the familiar object and the novel object later in the test phase. Kindled rats, on the other hand, did not suffer forgetting even when they were subjected to the retrieval phase, as they spent significantly longer times exploring the novel rather than the familiar object in the test phase. Therefore, RIF was not observed in the kindling group. These findings indicate impaired retrieval-induced forgetting in kindled rats, which may be suggestive of a deficit in the inhibitory control of memory.

## 1. Introduction

Retrieval-induced forgetting refers to the paradoxical finding wherein the act of remembering some information leads to the forgetting of other related, but non-retrieved information [1]. You may experience such forgetting when, for example, you encounter someone that you recognize, but you are unable to recall their name, even though it is “on the tip of your tongue”. Anderson and colleagues demonstrated this memory phenomenon with the retrieval-practice paradigm [1,2]. Subsequently, the effect has been shown to occur in a variety of experimental contexts, including category recognition [3], semantic relations [4], propositional knowledge [5], episodic memory [6], eyewitness [7], social [8], medical decision-making [9], and foreign language acquisition [10].

Typically, retrieval-induced forgetting is studied using the retrieval-practice paradigm [2], which in its basic form involves three phases: study, retrieval-practice, and test. In the study phase, subjects study a series of category-item pairs (e.g., fruit–apple, sport–tennis). In the practice phase, half of the items from half of the categories are practiced. In the test phase, all of the studied items (including those from both practiced and non-practiced categories) are tested for recall. As expected, practiced items from practiced categories are recalled best because of the additional practice, and all unpracticed items are less likely to be recalled when compared to practiced ones. However, unpracticed items belonging to practiced categories are less likely to be recalled compared to unpracticed items belonging to unpracticed categories. It is this impaired recall of related, but unpracticed items that is referred to as retrieval-induced forgetting.

Active inhibition of competing information during retrieval is the most widely accepted explanation for retrieval-induced forgetting [11,12]. In order to render desired information more accessible, related and potentially interfering information must be selected against, or inhibited.

One of the populations that are known to have impaired inhibitory control of executive functions is epilepsy patients [13,14]. Epilepsy is a disorder that is characterized by a persistent brain susceptibility to recurrent, unprovoked seizures, due to abnormally discharging central nervous system neurons. This chronic disorder is often associated with cognitive, neuropsychiatric, and behavioral comorbidities [15,16], in the form of depression [17], anxiety [18], migraines [19], autistic-like behavior [20], learning difficulties [21], and memory deficits [22,23]; features that are often perceived by the patients to be more detrimental to the quality of life than the seizures themselves [15].

Memory problems in epilepsy are the most commonly reported cognitive problems [24], found in about 30% of epilepsy patients [25], affecting both children and adults [26], and often represent a major management challenge [27]. The mechanisms underlying memory impairment in epilepsy have been studied extensively, and have been linked to deficits in executive functions [14].

Surprisingly, and to the best of our knowledge, there are no published studies on retrieval-induced forgetting in epilepsy. The purpose of this study is to determine whether epilepsy is associated with normal levels of retrieval-induced forgetting.

We attempted to demonstrate retrieval-induced forgetting in rats in a kindling model of epilepsy, using a modified spontaneous object recognition test.

Kindling was induced chemically by pentylenetetrazole, a GABA-A receptor antagonist known to cause a persistent increase in seizure liability with minimal neuronal damage [28]. In order to demonstrate retrieval-induced forgetting, we employed a modified version of the object recognition test that was proposed by Yamada and colleagues [29].

First, rats were presented with two different objects (not identical) in the sample phase, to allow for memorization of more than one object in a specific context (as in the study phase in the retrieval-practice paradigm). Then, a group of rats were presented with familiar objects (retrieval phase), and another group were presented with novel objects (interference phase). The interference phase served as a control for the retrieval phase. Finally, all rats were presented with a familiar object and a novel one in the test phase.

## 2. Materials and Methods

### 2.1. Animals

Sixty male Long-Evans rats aged 12–16 weeks (250–300 g) were individually housed in wire cages and were provided with food and water ad libitum. The animals were kept under controlled temperature and humidity conditions, in a 12-h light/dark cycle (lights on/off at 6:00/18:00 h).

All experimental procedures had been carried out from 08:00 to 16:00 h, and were approved by the Research Ethics Committee of the Arabian Gulf University, Manama, Bahrain (permit code: 56-PI-02/16). The animals have been randomly assigned to two groups: saline (*n* = 27) and kindling (*n* = 33).

### 2.2. Kindling

Kindling was induced following previously described methods [30,31]. Rats in the kindling group (*n* = 33) were injected with pentylenetetrazole dissolved in warm 0.9% saline intraperitoneally at a dose of 35 mg/kg in a volume of 10 mL/kg, on three non-consecutive days of the week. Following the injections, the animals were observed individually for thirty minutes within their glass observation cages (35 cm × 35 cm × 35 cm).

The ictal behaviors of the animals were evaluated according to a modified Racine’s score [32]; stage 1: sudden behavioral arrest and/or motionless staring; stage 2: facial jerking with muzzle or muzzle and eye; stage 3: neck jerks; stage 4: clonic seizure in a sitting position; stage 5: convulsions including clonic and/or tonic–clonic seizures while lying on the belly and/or pure tonic seizures; stage 6: convulsions including clonic and/or tonic–clonic seizures while lying on the side and/or wild jumping. A total of five generalized seizures were required to complete kindling. One week after the last pentylenetetrazole injection, these rats received a final challenge dose of pentylenetetrazole at a dose of 35 mg/kg to check whether their susceptibility to the convulsant was persistent. Only rats that had tonic–clonic seizures (stage 5–6) following the challenge dose were used in the remainder of the study (*n* = 27).

Rats in the saline group (*n* = 27) served as a control for the kindling group, and were injected with 0.9% saline intraperitoneally instead of pentylenetetrazole in a similar protocol as mentioned above, including a final injection 1 week after the final injection session. For both groups, behavioral testing was conducted on day 7 after the final challenge injection.

### 2.3. Modified Spontaneous Object Recognition Test

Retrieval-induced forgetting was tested using a previously described method [29]. An open field measuring 90 cm × 90 cm × 45 cm made of polyvinylchloride was used, and its walls and floor were gray. The objects used were yellow rubber duck (10 cm × 14 cm × 8 cm), purple metal box (10 cm × 8 cm × 6 cm), black cylinder of cast metal (5 cm in diameter, 16 cm in height), white plastic bottle (4 cm in diameter, 14 cm in height), green plastic cone (4 cm in diameter, 7 cm in height), a blue can of soda (3 cm in diameter, 5 cm in height), a small red plastic car (12 cm × 4 cm × 6 cm), and a tower of red, green, and yellow Lego bricks (6 cm × 6 cm × 18 cm). All of the objects were fixed on the floor using a double-sided adhesive tape to prevent them from being moved by the rats. Objects were rotated in a counterbalanced way to ensure all objects were employed equally in all 3 phases of the test, and all asymmetrical objects were presented in the same way. A video camera was suspended above the open field.

Rats received handling (5 min/day) and were allowed individually to explore the open field (with no objects placed) freely for habituation (10 min/day) for 3 days prior to the recognition task.

**Figure 1:**
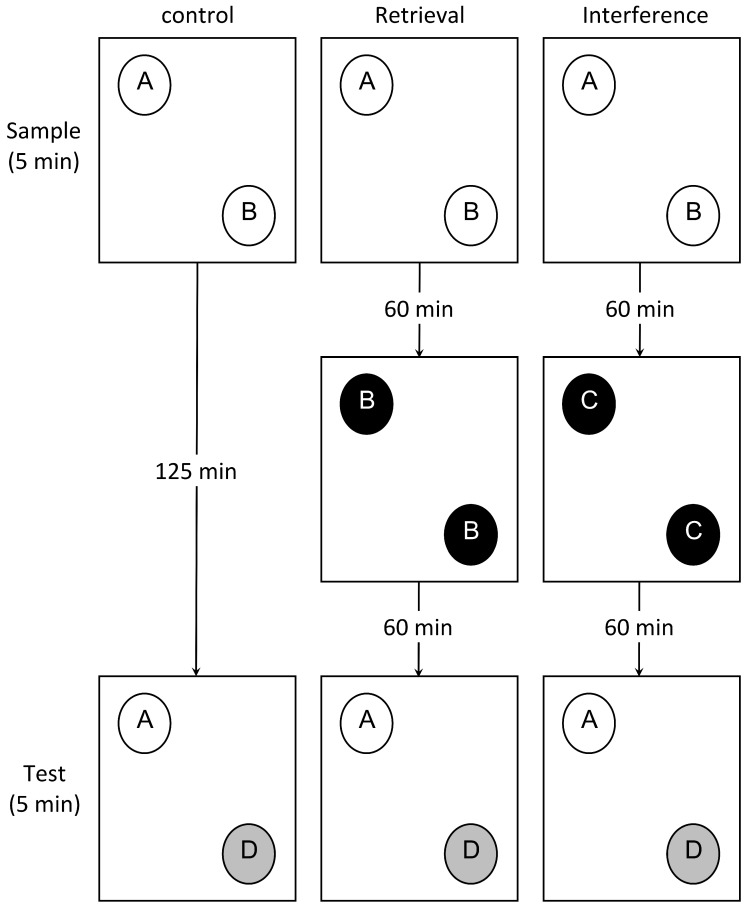
Modified object recognition test. In the sample phase, two different objects (A, B) were placed. One of these objects is replaced by a novel object in the test phase (A, D). For the retrieval group, two identical, related objects (B, B) were introduced in the delay period. For the interference group, two identical, unrelated objects (C, C) were introduced in the delay period. The animals were allowed to explore objects in each phase for 5 min, with a delay period (125 min) between the sample phase and the test phase.

The test consisted of a sample phase, retrieval or interference phase, and a test phase with 5-min exploration period and 60-min delay period inserted between the phases (Figure 1). Rats from each group (kindling and saline) were further divided into 3 groups: control (*n* = 9), retrieval (*n* = 9), and interference (*n* = 9) groups.

In the sample phase, rats were allowed to explore the field in which two different objects (A, B) were placed in the corner of the open field, for five minutes, followed by a delay period of 60 min in which rats were taken to their home cages. Following the sample phase, rats in the retrieval and the interference groups were introduced into the field again. The retrieval group were presented with two identical objects (B, B), which were familiar and identical to one of the objects presented in the sample phase. The interference group were presented with two identical objects (C, C) that were novel to the rats. Finally, in the test phase, rats were presented with two different objects (A, D), one of which was familiar and identical to the one presented in the sample phase, and the other was novel.

After each phase, the floor of the open field and the objects were sprayed with 70% ethanol, and then wiped with a damp cloth. All phases of the task were video-recorded, and time spent exploring each object was scored by two separate experienced observers viewing the rats’ behavior. Exploration was defined as the rat’s nose being directed toward the object at a distance less than or equal to 2 cm. Climbing onto the object or chewing it were not considered to be explorative behaviors.

### 2.4. Statistical Analysis

Differences in the amount of exploration between familiar and novel objects in the test phase were evaluated using the Student’s *t*-test. A discrimination ratio was calculated by dividing the amount of exploration of the novel object by the total amount of object exploration in the test phase, and the group factor was analyzed by a one-way analysis of variance (ANOVA), followed by post hoc analysis using Tukey’s honestly significant difference (HSD) test with alpha set at 0.05. The total amount of object exploration in seconds in the sample and test phases is reported as Mean ± SEM, followed by a comparison using a one-way analysis of variance (ANOVA). Statistical analysis was conducted using SPSS package (version IBM SPSS Statistic 23, Chicago, IL, USA).

## 3. Results

### 3.1. Presence of Retreival-Induced Forgetting Phenomenon in Saline-Treated Rats

**Figure 2:**
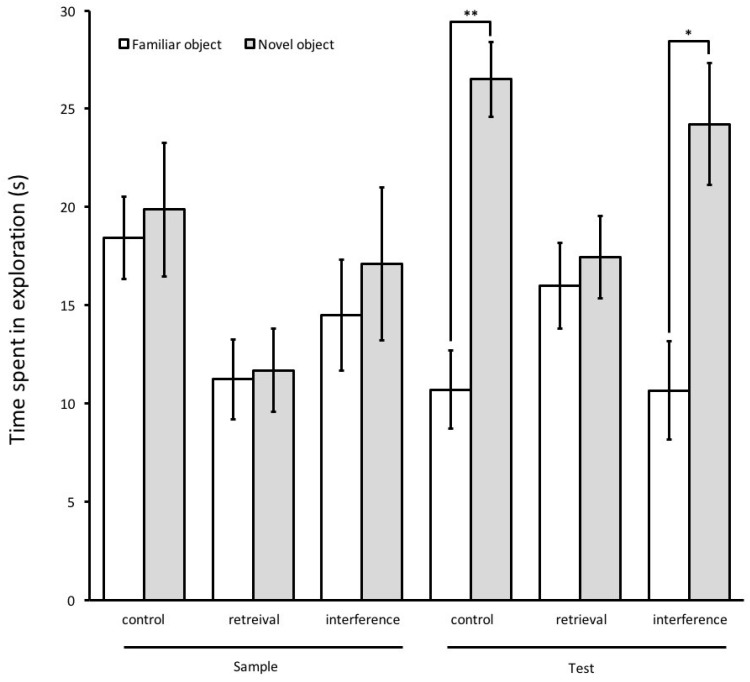
Time spent exploring objects (saline group). During the test phase, saline-treated rats in the retrieval group explored both objects similarly, while rats in control and interference groups explored the novel object more than the familiar one. The total amount of object exploration in seconds is shown as Mean ± SEM. * *p* < 0.05, ** *p* < 0.01.

**Figure 3:**
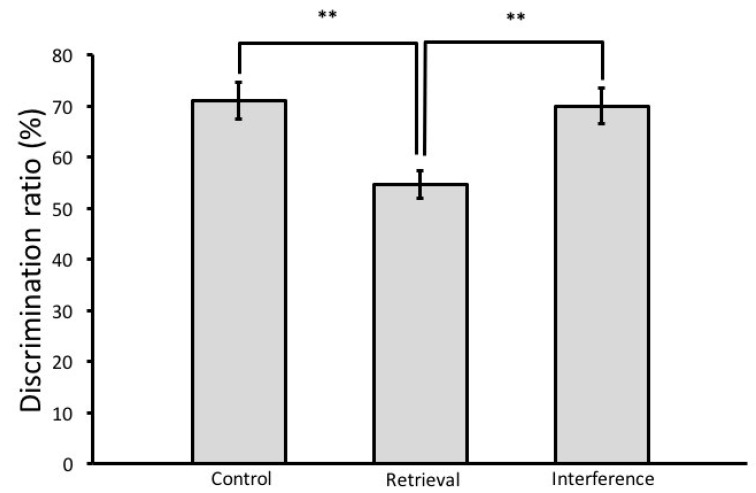
Discrimination ratio in the test phase (saline group). The discrimination ratio was calculated by dividing the total amount of exploration of the novel object by the total amount of object exploration. The retrieval group had significantly lower discrimination ratio than that of the control and the interference groups. Mean ± SEM is shown. ** *p* < 0.01.

In the sample phase, there were no significant differences in the exploration time of objects A and B, which indicates that animals in all groups explored both objects equally. In the test phase, the exploration time of the novel object was significantly longer than that of the familiar object in the control group (*t*-test, *t*(16) = 3.65, *p* = 0.001) and in the interference group (*t*-test, *t*(16) = 2.13, *p* = 0.024). Rats subjected to the retrieval phase, however, explored both objects almost equally in the test phase (*t*-test, *t*(12) = −1.06, *p* = 0.153), indicating that they could not discriminate between the familiar and the novel object (Figure 2). Analysis of the discrimination ratio in the test phase revealed a significant main effect of group (ANOVA, F(2,26) = 5.685, *p* = 0.01). A post hoc Tukey HSD test showed that the discrimination ratio of the retrieval group is significantly lower than that of the control (*p* = 0.017) and the interference group (*p* = 0.024) (Figure 3). The total amount (Mean ± SEM) of object exploration in the test phase was 18.6 ± 2.27 s in the control group, 16.7 ± 3.54 s in the retrieval group, and 17.44 ± 3.54 s in the interference group. Analysis of the total exploration time showed no significant differences between the three groups in the test phase (ANOVA, F(2,53) = 1.074, *p* = 0.349).

### 3.2. Absence of Retreival-Induced Forgetting Phenomenon in Kindled Rats

**Figure 4:**
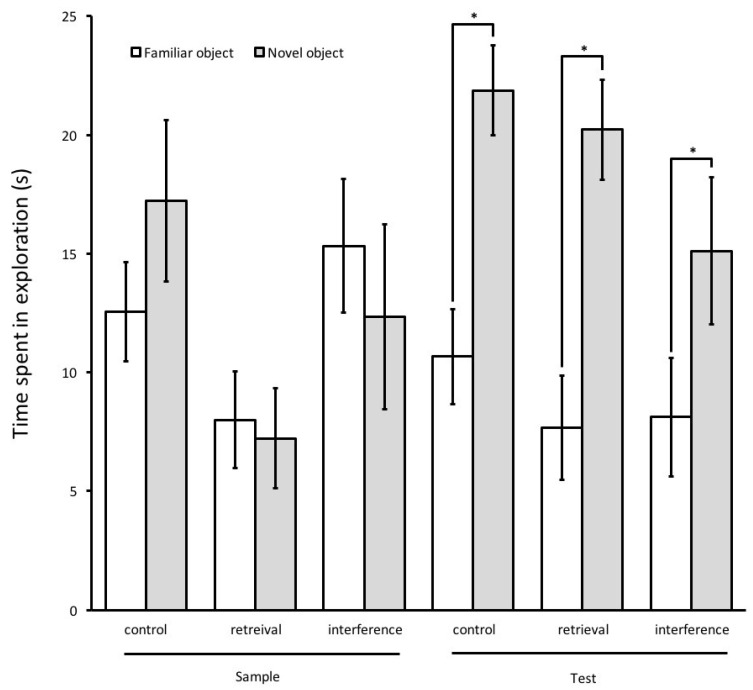
Time spent exploring objects (kindling group). In all three groups during the test phase, kindled rats explored the novel object more than the familiar one. The total amount of object exploration in seconds the test phase is reported as Mean ± SEM. * *p* < 0.05.

**Figure 5:**
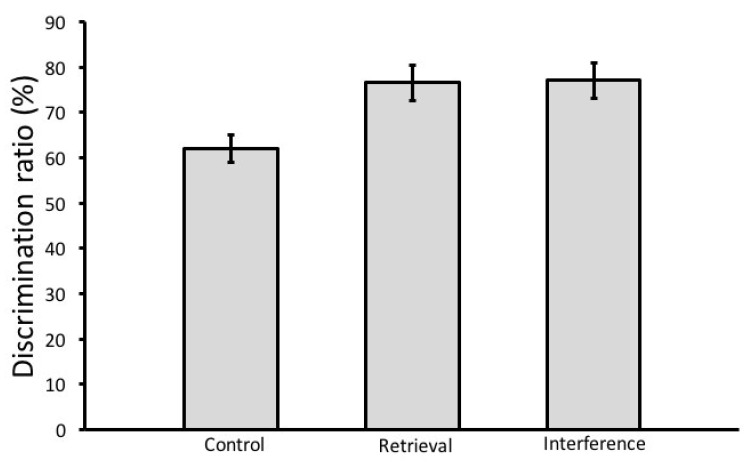
Discrimination ratio in the test phase (kindling group). The discrimination ratio was calculated by dividing the total amount of exploration of the novel object by the total amount of object exploration. No significant difference was found between the three groups. Mean ± SEM is shown.

Kindled rats explored both objects equally in the sample phase, in all three groups, while in the test phase they spent significantly longer times exploring the novel object than the familiar object for all three groups: control group (*t*-test, *t*(16) = 3.42, *p* = 0.001), interference group (*t*-test, *t*(16) = 1.84, *p* = 0.041), and retrieval group (*t*-test, *t*(16) = 2.98, *p* = 0.004), (Figure 4). With regard to the discrimination ratio, no significant group effect was observed in the test phase (ANOVA, F(2,26) = 0.601, *p* = 0.556) (Figure 5). The total amount (Mean ± SEM) of object exploration in the test phase was 16.2 ± 2.1 s in the control group, 13.9 ± 2.5 s in the interference group, and 13 ± 2.36 s in the interference group. Analysis of the total exploration time showed no significant differences between the three groups in the test phase (ANOVA, F(2,53) = 0.519, *p* = 0.598).

### 3.3. No Differences in Exploratory Behavior between Saline-Treated Rats and Kindled Rats

Both groups had similar total exploration times in the sample phase of the modified object recognition task (ANOVA, F(1,53) = 0.413, *p* = 0.837), and in the test phase (ANOVA, F(1,53) = 0.556, *p* = 0.733).

## 4. Discussion and Conclusions

This study was conducted in order to investigate whether kindled rats demonstrate normal levels of retrieval-induced forgetting. Kindling is a well-established experimental model of epilepsy [33]. It can be broadly defined as the process of inducing behavioral and electrographic seizures by repeated electrical or chemical stimulation, which results in network hyperexcitability and reduced seizure threshold. Many of the pathophysiological changes that are observed in epilepsy patients, are also observed in kindled animals [34]. These changes include neurodegeneration [35], reactive gliosis [36], and dysregulation in the expression of effector immediate early genes processes likely underlying epileptogenesis [34,37]. Pentylenetetrazole-induced kindling in particular, leads to reductions in GABAergic transmission, and/or increase in glutamatergic transmission, which results in an imbalance between excitatory and inhibitory tone. These changes, in addition to reductions in cortical and hippocampal dopaminergic and serotonergic neurotransmission are believed to lead to memory impairment in kindled animals [38].

In the present study, kindled rats demonstrated reduced levels of retrieval-induced forgetting. First, retrieval-induced forgetting was demonstrated in the saline group when rats subjected to the retrieval phase failed to discriminate between the familiar and novel objects in the test phase, indicated by similar exploration times of both objects and a low discrimination ratio compared to the control and interference groups. These findings suggest that the retrieval of an object impairs later recall of related but non-retrieved object, similar to what is observed in the typical retrieval-practice paradigm in humans. Kindled rats, however, spent more time exploring the novel object in the test phase in all three groups, with no differences in the discrimination ratio. Therefore, the insertion of a retrieval phase did not seem to alter later recall of related, but non-retrieved object. These findings suggest that kindled rats do not show normal levels of retrieval-induced forgetting.

The insertion of an additional phase (the retrieval phase) between the sample and the test phases posed the possibility that rats might be less motivated to perform in the following test phase. This was ruled out by inserting an interference phase, which served as a control for the retrieval phase. Rats in the interference group were presented with two unrelated, novel objects before the test phase. In the test phase, no significant differences in the total exploration time were found between the retrieval and the interference groups.

A number of rats completed kindling, but did not show generalized tonic–clonic seizures after the final challenge dose (*n* = 6), and therefore were excluded from behavioral testing. It is unclear whether these rats would have shown normal levels of retrieval-induced forgetting. Research shows that variations in kindling models may produce significantly different cognitive outcomes [30,34]. Therefore, findings of this study should be interpreted cautiously, as they are limited to pentylenetetrazole kindling, and do not necessarily suggest that other methods of kindling would produce similar outcomes, or that the degree of retrieval-induced forgetting is correlated to the severity of brain changes or seizure threshold after kindling.

Anxiety in rats may inhibit exploratory behavior [39]. The administration of pentylenetetrazole is reported by some studies to produce an anxiolytic effect [40,41], while other studies reported higher anxiety levels [30,42]. A limitation of this study is that anxiety was not assessed independently. Nevertheless, exploratory behavior was not affected by kindling, as the total exploration time was similar to that of the saline-treated rats in the sample phase and in the test phase. Also, object recognition memory was not affected by kindling, as both groups were able to recognize the familiar object in the control phase of the task, findings similar to what is reported by Hoeller and colleagues [43].

Our findings support the inhibitory account of retrieval-induced forgetting. As mentioned, it is widely accepted that retrieval-induced forgetting results from the active inhibition of competing, interfering information [11,12]. In contrast, non-inhibitory accounts of retrieval-induced forgetting, such as blocking-based accounts [11,44], argue that practiced items are simply strengthened and occupy a “response channel” in memory, effectively blocking unpracticed, related information from occupying the same response channel (see Anderson [1] for a review of non-inhibitory theories of retrieval-induced forgetting). However, recent evidence from neuroimaging studies [45,46] and electrophysiology [47], along with several demonstrations that are difficult for non-inhibitory accounts to explain (e.g., forgetting still occur even with failed retrieval attempts where nothing is retrieved, and therefore nothing is strengthened [48,49]), make the inhibitory account the best-supported explanation for retrieval-induced forgetting [50].

Inhibitory function is a core component of the cognitive processes that are necessary for organization, planning, sustained attention, and executing goal-directed tasks, often referred to as executive functions [51]. Based on the inhibitory account of retrieval-induced forgetting, many researchers have predicted that populations that are believed to have inhibitory deficits and poor executive functions would show less or even no such forgetting. These populations include individuals with clinical depression [52], schizophrenia [53], frontal lobe damage [54], Alzheimer’s disease [55], attention deficit hyperactivity disorder [50], young children [56,57], and old adults [58]. Interestingly, only individuals with clinical depression and attention deficit hyperactivity disorder have been shown to have reduced levels of retrieval-induced forgetting.

The fact that the majority of populations with well-established inhibitory deficits may still exhibit normal levels of retrieval-induced forgetting has led some to argue that the inhibition underlying retrieval-induced forgetting is of a more implicit, automatic type, and should be distinguished from other types of inhibition that are involved in executive functions and more demanding tasks, where the inhibition is intentionally-controlled, and the instruction to forget is explicit, such as think/no-think or Stroop-like procedures [50]. According to this view, inhibitory control is not an all-or-nothing matter, and individuals may vary in their abilities to control attention. Therefore, people known to have inhibitory deficits in demanding tasks, such as executive function tasks, may still have adequate levels of inhibitory control in less demanding tasks, like retrieval-practice tasks. In the case of attention deficit/hyperactivity disorder, it appears that impaired inhibitory control does not only affect executive functions, but also the covert, unconscious cognitive operations related to retrieval-induced forgetting. It is not possible, however, to determine whether kindling impairs implicit or explicit inhibition based on the findings of this study. 

While forgetting is often perceived as a failure of memory, many have argued that retrieval-induced forgetting is of beneficial value in many situations, and critical for the efficient and adaptive functioning of memory [59,60]. Research shows that autobiographical memories are susceptible to retrieval-induced forgetting [61,62]. Moreover, negative autobiographical memories (related to traumatic events, for example) have been shown to be more susceptible to retrieval-induced forgetting than positive ones [63,64]. In this context, retrieval-induced forgetting helps maintain a positivity bias that keeps intrusive negative thoughts from being consciously retrieved. It comes as no surprise that deficits in retrieval-induced forgetting have been linked to depression [52], posttraumatic stress disorder [65], social phobia [66] and anxiety [67]. As mentioned previously, epilepsy is associated with several neuropsychiatric comorbidities, including depression [18]. Deficits in the inhibitory control of memory in epilepsy may lead to a reduction in the levels of retrieval-induced forgetting, as our findings suggest. That, in turn, may play a role in the increased propensity for epilepsy patients to experience intrusive negative memories, depression, and anxiety.

Findings of this study suggest a tendency for kindled rats to demonstrate impaired levels of retrieval-induced forgetting, which could represent an underlying deficit in the inhibitory control of memory. However, this data is not sufficient to conclude that a similar tendency is to be found in humans. Further research is recommended to explore retrieval-induced forgetting in epilepsy patients.
